# Exercise‐Induced Cardiac Lymphatic Remodeling Mitigates Inflammation in the Aging Heart

**DOI:** 10.1111/acel.70043

**Published:** 2025-03-13

**Authors:** Kangsan Roh, Haobo Li, Rebecca Nicole Freeman, Luca Zazzeron, Ahlim Lee, Charles Zhou, Siman Shen, Peng Xia, Justin Ralph Baldovino Guerra, Cedric Sheffield, Timothy P. Padera, Yirong Zhou, Sekeun Kim, Aaron Aguirre, Nicolas Houstis, Jason D. Roh, Fumito Ichinose, Rajeev Malhotra, Anthony Rosenzweig, James Rhee

**Affiliations:** ^1^ Corrigan Minehan Heart Center and Cardiology Division, Massachusetts General Hospital, Harvard Medical School Boston Massachusetts USA; ^2^ Department of Anesthesia Critical Care, and Pain Medicine, Massachusetts General Hospital Boston Massachusetts USA; ^3^ Scripps Research Institute, Department of Chemistry California La Jolla USA; ^4^ Department of Integrative Biotechnology Sungkyunkwan University Suwon Republic of Korea; ^5^ Wellman Center for Photomedicine, Massachusetts General Hospital Boston Massachusetts USA; ^6^ Center for Systems Biology, Massachusetts General Hospital Boston Massachusetts USA; ^7^ Stanley and Judith Frankel Institute for Heart and Brain Health, University of Michigan Medical Center Ann Arbor Michigan USA; ^8^ Tufts University School of Medicine Boston Massachusetts USA; ^9^ Edwin L. Steele Laboratories, Department of Radiation Oncology, Massachusetts General Hospital Boston Massachusetts USA; ^10^ Center for Advanced Medical Computing and Analysis (CAMCA), Massachusetts General Hospital Boston Massachusetts USA

**Keywords:** exercise, heart, lymphangiogenesis, lymphatic endothelial cells, lymphatic vessels, VEGF‐C, ventricular remodeling

## Abstract

The lymphatic vasculature plays essential roles in fluid balance, immunity, and lipid transport. Chronic, low‐grade inflammation in peripheral tissues develops when lymphatic structure or function is impaired, as observed during aging. While aging has been associated with a broad range of heart pathophysiology, its effect on cardiac lymphatic vasculature has not been characterized. Here, we analyzed cardiac lymphatics in aged 20‐month‐old mice versus young 2‐month‐old mice. Aged hearts showed reduced lymphatic vascular density, more dilated vessels, and increased inflammation and fibrosis in peri‐lymphatic zones. As exercise has shown benefits in several different models of age‐related heart disease, we further investigated the effects of aerobic training on cardiac lymphatics. Eight weeks of voluntary wheel running attenuated age‐associated adverse remodeling of the cardiac lymphatics, including reversing their dilation, increasing lymph vessel density and branching, and reducing perilymphatic inflammation and fibrosis. Intravital lymphangiography demonstrated improved cardiac lymphatic flow after exercise training. Our findings illustrate that aging leads to cardiac lymphatic dysfunction, and that exercise can improve lymphatic health in aged animals.

## Graphical abstract

Exercise improves cardiac lymphatic architecture and function in the aged mouse. Aged hearts show reduced lymphatic vascular density, more dilated vessels, and increased peri‐lymphatic inflammation and fibrosis. Exercise training of the aged mouse increases lymph vessel density and sprouting, reverses their dilation, reduces inflammation and fibrosis, and restores epicardial lymphatic flow.

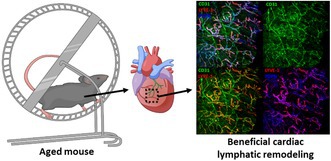

**Abbreviations**
AAV: adeno‐associated virus
aExTr: aged exercise‐trained
ANOVA: analysis of variance
aSed: aged sedentary
CD: cluster of differentiation
DAPI: 4′,6‐diamidino‐2‐phenylindole
ExTr: exercise‐trained
FITC: fluorescein isothiocyanate
LEC: lymphatic endothelial cell
LYVE‐1: lymphatic vessel endothelial hyaluronan receptor 1
MI: myocardial infarction
ns: not significant
PDPN: podoplanin
PLV: popliteal lymphatic vessels
RELN: reelin
Sed: sedentary
VE‐cadherin: vascular endothelial cadherin
VEGF: vascular endothelial growth factor
VEGFR: vascular endothelial growth factor receptor
WGA: wheat germ agglutinin

## 1 Introduction

Lymphatic vessels return extravasated interstitial fluid into circulating blood to maintain interstitial pressure, facilitate antigen clearance, and resolve inflammation (Huggenberger et al. 2011; Kataru et al. 2009). Perception of the lymphatic system has evolved from one of largely passive flow to an appreciation of its essential and dynamic roles in total body fluid balance and transport of critical mediators of metabolism and inflammation (Davis et al. 2000; Mehlhorn et al. 2001). Diminished lymphatic function is observed in many disease states, such as infection and obesity, and as a complication of cancer therapy, with ensuing secondary lymphedema and chronic fibrosis (Garza et al. 2017; Kim et al. 2015; Kunkler et al. 2015; Stevens et al. 2005). Cardiac lymphatic dysfunction was identified in various disease states over 50 years ago (Bradham and Parker 1973; Bradham et al. 1970), and has since been observed in hypertension (Chachaj et al. 2018; Machnik et al. 2009), dyslipidemia (Brakenhielm and Alitalo 2019; Lim et al. 2013), atherosclerosis (Martel et al. 2013), and myocardial infarction (Henri et al. 2016; Klotz et al. 2015; Vuorio et al. 2017). Stimulation of lymphangiogenesis with vascular endothelial growth factor (VEGF)‐C administration following heart injury improves cardiac recovery and function (Klotz et al. 2015).

Recent studies have suggested that exercise can improve peripheral lymphatic function. Exercise enhances the frequency and vigor of lymphatic pumping and decreases lymphatic vessel leakiness, indicating a reversal in the pathology of the lymphatic endothelium. Aerobic exercise reverses obesity‐induced lymphatic dysfunction and decreases perilymphatic inflammation independent of weight loss (Baumann et al. 2018; Bok et al. 2016; Hespe et al. 2016). Exercise restores fluid uptake and lymph transport in obesity‐related lymphatic impairment and augments the transport of immune cells from peripheral tissues to regional lymph nodes via increased chemokine expression on lymphatic endothelial cells (LECs) (MartIn‐Fontecha et al. 2003). In breast cancer survivors with lymphedema, exercise improves lymph flow and protein resorption (Mortimer 1995) and mitigates lymphedema and its symptomatology (Brennan and Miller 1998; Schmitz et al. 2009; Schmitz et al. 2010).

Lymphatic architecture across different organ systems changes during aging (Petrova and Koh 2018). Aging‐related pathophysiologic changes include enlarged lymphatic diameter, decreased pumping activity, and diminished response to pathogens (González‐Loyola and Petrova 2021; Kataru et al. 2022; Zolla et al. 2015). However, it is unknown how cardiac lymphatic vessels remodel with age, and how this might affect microenvironments within the heart. Here, we have characterized alterations in the cardiac lymphatic vessels in old mice. Since exercise confers a broad range of cardiovascular benefits (Hastings, Castro, et al. 2024; Hastings et al. 2022; Hastings, Zhou, et al. 2024; Lerchenmuller et al. 2022; Li et al. 2020; Roh et al. 2016; Roh et al. 2020; Trager et al. 2023), we investigated whether it improves lymphatic structure and perilymphatic markers of health in cardiac aging. We found that the hearts of 20‐month‐old mice had lymphatic vasculature that was less dense and more dilated than those of 2‐month‐old mice. After 8 weeks of aerobic exercise training, aged hearts exhibited lymphangiogenesis, improved vessel density, and decreased vessel dilation. Enhanced cardiac lymphatic flow in aged, exercised mice occurred in parallel with improved cardiac function after 6 weeks of treadmill running. Notably, perilymphatic collagen deposition and immune cell infiltration, both hallmarks of cardiac aging, were reduced after exercise. Thus, exercise training restores lymphatic function and immune homeostasis in the aged heart.

## 2 Materials and Methods

### 2.1 Mice

All animal studies were approved by the Massachusetts General Hospital Institutional Animal Care and Use Committees (IACUC, protocol numbers 2015 N000029, 2022 N000209). C57BL/6 male mice were obtained from either The Jackson Laboratory (young 2‐month‐old and aged 20‐month‐old mice) or the National Institute on Aging (aged 22‐month‐old mice). All analyses were performed in a blinded manner.

*Exercise training protocols*.

Mice were randomly assigned to sedentary control or exercise groups. Voluntary wheel running was performed for eight consecutive weeks using previously published methods (Lerchenmuller et al. 2022). Briefly, mice were individually housed in plexiglass cages (36 L x 20 W x 15H cm) that each contained a stainless steel running wheel (diameter 11.4 cm; Mini‐Mitter, Starr Life Science, USA) connected to a sensor measuring wheel revolutions. Sedentary control mice were housed in identical polypropylene cages with no wheels. For cardiac lymphangiography studies, mice underwent moderate‐intensity treadmill running 5 days per week for six consecutive weeks. Treadmill running was done on an automated treadmill (Columbus Instruments) at a constant speed of 10 m/min at a 30° incline for one hour each day.

### 2.2 Immunofluorescence

After mice were euthanized, their hearts were perfused with ice‐cold PBS just prior to explantation. They were fixed with 4% paraformaldehyde (PFA) and then immersed in a blocking solution consisting of 10% normal donkey serum. Immunostaining was performed using primary antibodies, including anti‐LYVE‐1 (1:100, #11–034, Angiobio), anti‐LYVE‐1 (1:100, #MAB2125‐100, R&D SYSTEMS), anti‐VEGFR3 (1:100, #AF743, R&D SYSTEMS), anti‐VE‐Cadherin (1:100, # AF1002, R&D SYSTEMS), anti‐Ki‐67 (1:50, #14–5698‐82, eBioscience), anti‐CD31 (1:100, #557355, BD Pharmingen), anti‐CD3 (1:100, #100236, BioLegend), anti‐CD68 (1:100, #MCA1957, Biocompare), anti‐CD206 (1:100, #PA5‐46994, Invitrogen), anti‐ACTN2 (1:100, #14221‐1‐AP, Proteintech), anti‐COL1 (1:100, #NB600‐450, Novus Biologicals), anti‐Perilipin1 (1:100, #9349S, Cell Signaling), and anti‐WGA (1:200, W11261, Invitrogen). Secondary antibodies conjugated with Alexa Fluor 488, 594, and 647 were purchased from Jackson ImmunoResearch. DAPI staining (1:1000, #D1306, Invitrogen) was employed for visualizing the nuclei. All antibodies used in this study were validated for use in mice and their respective applications by the manufacturers.

### 2.3 Whole‐Mount Tissue Clearing and Imaging

Immunostained tissues were cleared with a simplified version of 3DISCO (Renier et al. 2014). The tissues underwent overnight incubation in 50% v/v tetrahydrofuran (THF, #401757, Sigma‐Aldrich). The samples were incubated for 1 h in 80% THF, twice for 1 h in 100% THF, and then in dichloromethane (#L090000, Sigma‐Aldrich). Following this, the samples were immersed in a 1:2 ratio mixture of benzyl alcohol (#305197, Sigma‐Aldrich) and benzyl benzoate (#B6630, Sigma‐Aldrich) and stored in glass vials at room temperature until imaging. Confocal images were captured using a Leica TCS SP8 X Laser Confocal Microscope. The acquisition and processing of these images were performed using LAS X software (Leica). The high‐resolution images of the stained whole‐mount hearts were generated as maximum intensity projections from tiled z‐stack images, acquired at 5‐μm intervals across the entire thickness of the tissues. Quantitative imaging analyses were conducted in a blinded fashion. Each data point in the quantification of all confocal and whole‐mount images represents the average of at least three randomly selected microscopic fields per mouse.

### 2.4 Lymphatic Diameter and Vessel Density

Heart tissues were fixed in 4% paraformaldehyde and processed for whole‐mount immunostaining using anti‐LYVE1 and anti‐CD31 antibodies to specifically label cardiac lymphatic endothelial cells. Lymphatic diameters were analyzed with LAS X software, measuring at least three distinct regions along each vessel. The mean of these measurements was calculated to represent the diameter of each vessel. To determine the group‐level value, the average diameter of a minimum of three vessels per heart was calculated, and the pooled data from all samples were used for statistical analysis. Lymphatic vessel density was quantified by counting the number of LYVE1+ VEGFR3+ epicardial lymphatic vessels within a defined area (1.32 mm^2^) of each heart. Each density data point represents the average number of vessels from at least three separate regions of the epicardium per mouse.

### 2.5 Lymphangiography

Mice were anesthetized (100 mg/kg ketamine‐10 mg/kg xylazine) and injected interstitially with fluorescent dye (4 μL 2% FITC‐dextran, MW: 2 M Da, Thermo Fisher Scientific) into the left footpad. To expose the popliteal collecting lymphatic vessels, a small incision was made in the left hindlimb as previously described (Liao et al. 2011; Liao et al. 2014). Time‐lapse images of the exposed vessels were acquired using an inverted fluorescence microscope, capturing 360 frames at 80‐ms intervals. Three sets of measurements were obtained from each mouse at 15‐min intervals, and the average value was used to determine lymphatic contraction. Lymphatic pumping frequency and strength were analyzed using time‐lapse images processed through custom MATLAB scripts, based on the peak‐and‐valley method previously established (Liao et al. 2014). For peripheral lymphatic drainage measurements, FITC‐dextran was injected into the footpad of the left hindlimb of anesthetized mice. Popliteal, inguinal, and axillary lymph nodes were harvested 1.5 h post‐injection, and FITC fluorescence signals were captured from these draining lymph nodes (Leica confocal microscopy) and quantified (ImageJ). For intravital cardiac lymphangiography, mice were anesthetized with an intraperitoneal injection of ketamine (180 mg/kg) and fentanyl (0.13 mg/kg). After performing a tracheostomy, mice were paralyzed with rocuronium (1 mg/kg) and mechanically ventilated (TV 10 mL/kg, RR 110 breaths per minute, PEEP 2, FiO2 21%). A PE10 catheter was inserted in the carotid artery to monitor mean systemic blood pressure and heart rate. Body temperature was monitored with a rectal probe and maintained at 37°C with a heating pad. Following median thoracotomy and exposure of the heart, fluorescent dye (10 μL 5% FITC‐dextran, MW: 2 M Da, Thermo Fisher Scientific) was injected into the epicardium at the cardiac apex, followed by a 5‐min circulation period. Hearts were carefully excised, fixed in PFA, and prepared for imaging with a stereo microscope (SZX16, Olympus) to capture comprehensive views of the anterolateral epicardium. FITC signals were then acquired using an Olympus DP72 microscope and digital camera. Image acquisition and subsequent processing were conducted using ImageJ software, enabling the visualization and reconstruction of FITC‐dextran‐labeled epicardial lymphatic networks.

### 2.6 Quantitative Real‐Time PCR

RNA from tissues and cells was extracted using Trizol reagent (Invitrogen #15596018) with phase separation followed by column purification (Qiagen). Real‐time quantitative PCR was performed using SYBR green and standard amplification protocols. Expression levels were calculated using the ∆∆Ct method. Primer sequences are listed in Table S1.

### 2.7 Flow Cytometry

Hearts from aged C57BL/6 mice were harvested and immediately placed in cold Hank’s Balanced Salt Solution (#H6648, Sigma‐Aldrich). The tissue was finely minced into small fragments and enzymatically digested in 500 U/mL of collagenase type II solution (#LS004176, Worthington Biochemical Corp.) at 37°C with constant agitation for 30 minutes. The supernatant was removed, and 10% fetal bovine serum (#A5256701, Gibco) was added to neutralize enzymatic activity. The remaining tissue was subjected to two additional rounds of digestion using fresh collagenase solution. The resulting cell suspensions were pooled and filtered through a 70 μm cell strainer (#CLS352350, Corning). The cells were then centrifuged, washed with PBS, and treated with Red Cell Lysis buffer (#420301, BioLegend) according to the manufacturer’s protocol to remove residual erythrocytes. Isolated cells were resuspended in a 2% FBS solution and Fc‐blocked (BD Biosciences) on ice. Subsequently, cell suspensions were stained with anti‐CD45‐Alexa647 (#160303, BioLegend), anti‐CD31‐FITC (#102506, BioLegend), anti‐LYVE‐1‐Daylight 680 (#NB100‐725FR, Novus Biologicals), anti‐VEGFR‐3 (#BS‐1083R, Bioss), and LIVE/DEAD Cell Stain kit (#L34961, Invitrogen) and analyzed on an LSR Fortessa flow cytometer (BD Biosciences).

### 2.8 Statistical Analyses

Statistical testing was performed using Graphpad Prism 10.1.2. An unpaired, two‐tailed Student t‐test was used for comparisons between two groups, with a *p* < 0.05 considered statistically significant. When evaluating multiple groups, one‐way ANOVA was employed with post hoc Tukey's multiple comparison testing. The number of animals used for each group was determined based on total empirical data and the anticipated completeness of datasets, ensuring it was adequate to detect differences in experimental outcomes if present.

## 3 Results

### 3.1 Exercise Training in Young Mice Stimulates Peripheral Lymphatic Flow and Cardiac Lymphangiogenesis

**FIGURE 1:**
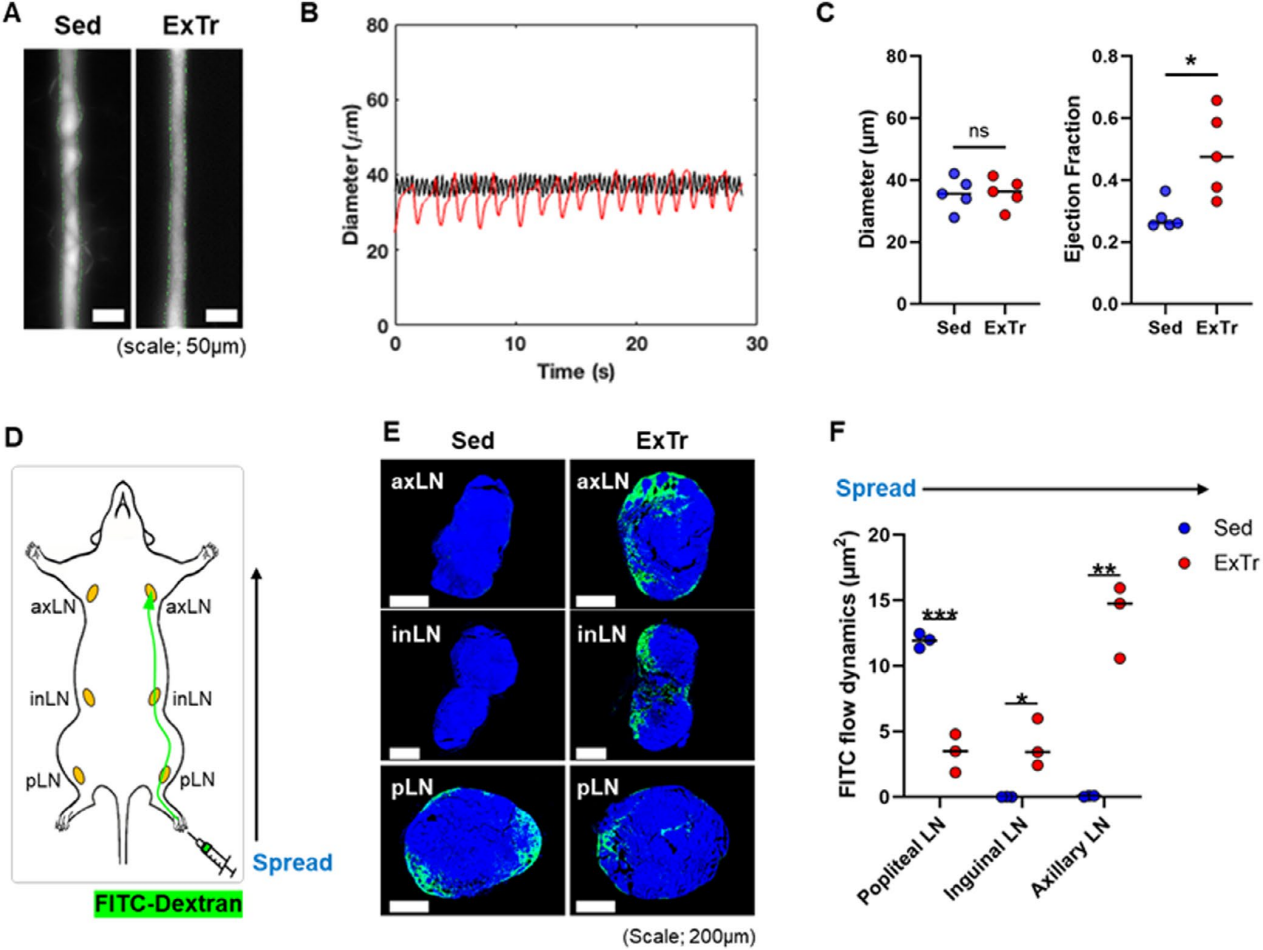
Exercise training increased ejection fraction of collecting popliteal lymphatic vessels (PLV) and enhanced lymph flow. (A) Representative intravital microscopy images of PLVs perfused with FITC‐dextran from sedentary (Sed) and exercise‐trained (ExTr) 2‐month‐old mice. (B) Representative lymphatic contraction curves in sedentary (Sed) control and exercise‐trained (ExTr) mice. (C) Quantification of lymphatic diameter and pumping (*n* = 5 per group). (D) Schematic diagram of lymphatic pumping and flow after FITC dextran injection into the foot pad. (E) Representative images of transport of FITC‐dextran through draining popliteal, inguinal, and axillary lymph nodes with (F) quantification of FITC in proximal to distal lymph nodes (*n* = 3 per group). *p < 0.05; **p < 0.01; ***p < 0.001; ns: Not significant.

Two‐month‐old C57BL/6 male mice underwent 8 weeks of voluntary wheel running (average daily distance per mouse = 6.26 km), which induces physiological cardiac growth and cardiomyogenesis (Li et al. 2022; Vujic et al. 2018). This exercise training (ExTr) resulted in increased ejection fraction in popliteal lymphatics measured with intravital microscopy (Figure 1A–C, Figure S1). Drainage of fluorescent tracer (FITC‐Dextran) injected into the foot pad of ExTr mice exhibited a much quicker transit time to the distant axillary lymph node, indicating more effective lymphatic pumping even at rest (Figure 1D–F). Conversely, sedentary mice demonstrated slower lymphatic transport from the foot pad, with most of the fluorescent marker remaining in the proximal popliteal lymph node over the same timeframe.

**FIGURE 2:**
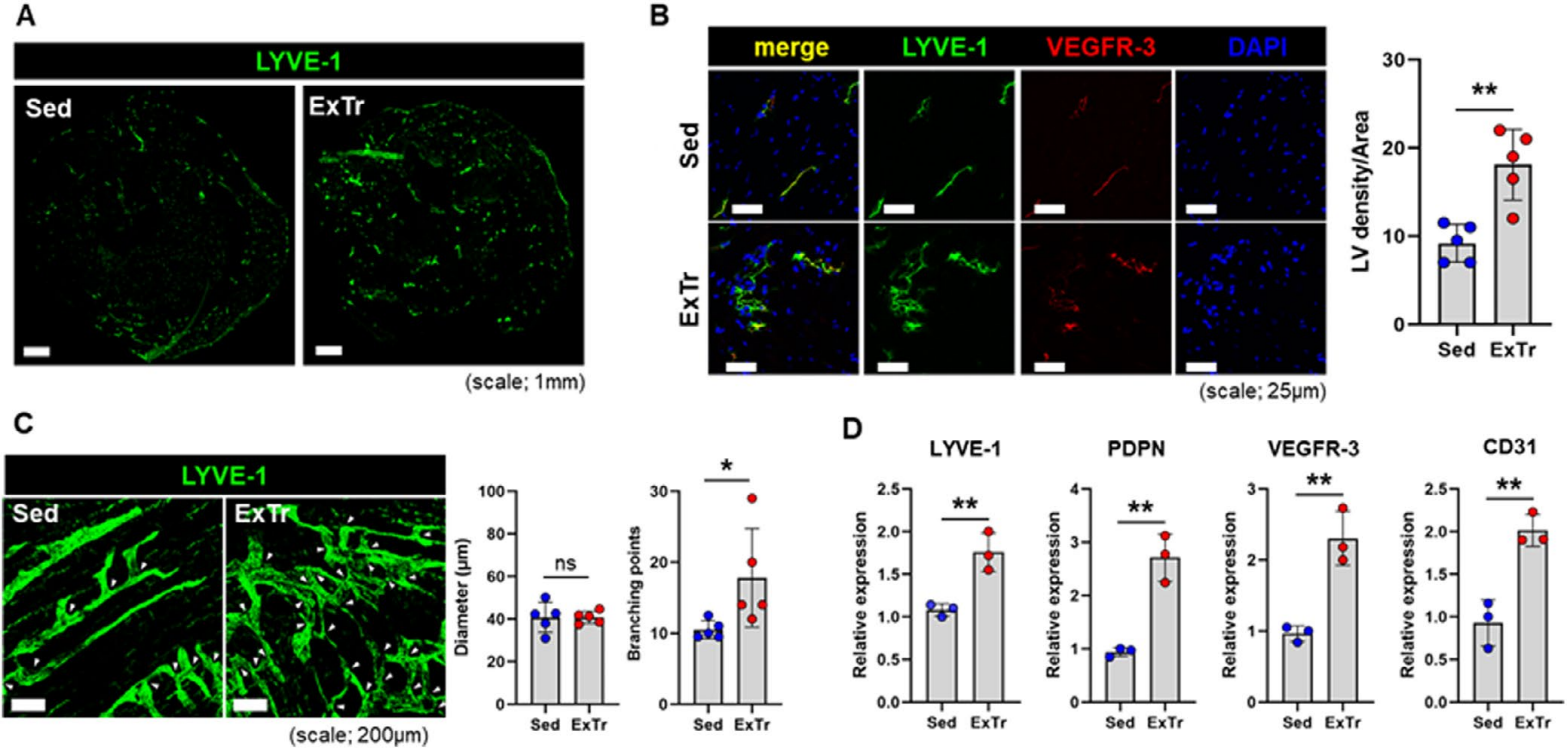
Exercise training induced cardiac lymphangiogenesis in young mice after 8 weeks of voluntary wheel running. (A) LYVE‐1 immunohistochemical staining of the lymphatic network in hearts from sedentary (Sed) control and exercise‐trained (ExTr) mice. (B) Confocal imaging for LYVE‐1 and VEGFR‐3 in heart tissues from sedentary control and exercised mice with quantification of cardiac lymphatic density (*n* = 5 per group). (C) Representative whole mount staining of LYVE‐1+ epicardial lymphatics from sedentary control and exercised mice with quantification of lymphatic diameter and branching points (white arrows) (*n* = 5 per group). (D) mRNA levels of CD31 and lymphatic markers LYVE‐1, PDPN, and VEGFR‐3 in sedentary control and exercised mice. *p < 0.05; **p < 0.01; ns: Not significant.

ExTr increased expansion of the cardiac lymphatic network as visualized by immunohistochemical staining for lymphatic vessel endothelial hyaluronan receptor 1 (LYVE‐1) in whole heart cross sections (Figure 2A). LYVE‐1 quantification under higher magnification showed a near doubling of lymphatic vessel density in the ExTr group (Figure 2B), as well as an increase in the number of vessels positive for VEGFR‐3, the receptor for VEGF‐C. While there was no difference in the diameter of cardiac lymphatic vessels in young mice after wheel running, there were more branching points of LYVE‐1+ lymphatic vessels (Figure 2C). Levels of mRNA of LYVE‐1, podoplanin (PDPN), and VEGFR‐3 as representative lymphatic endothelial cell markers were all significantly higher in the hearts of exercised mice (Figure 2D).

### 3.2 Aged Cardiac Lymphatics Demonstrate Dilation and Loss of Integrity

**FIGURE 3:**
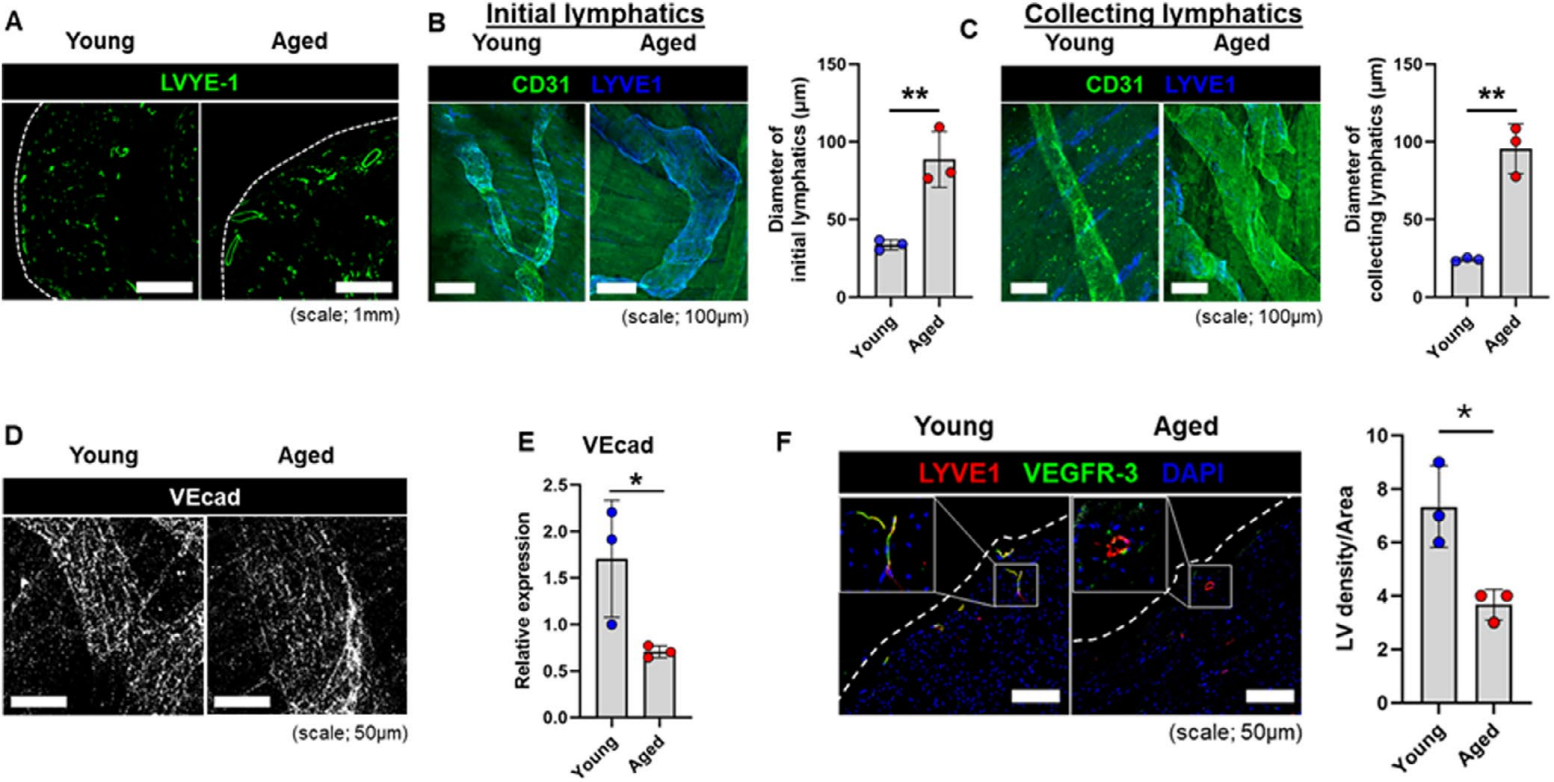
Aging caused dilation of cardiac lymphatics and impaired integrity of lymphatic vessels. (A) Confocal imaging and quantification of vessel diameter of LYVE‐1+ lymphatic vessels in hearts from young 2‐month‐old and aged 20‐month‐old mice. (B) Representative whole mount staining of CD31 + LYVE‐1+ initial lymphatics in the hearts from young and aged mice with quantification of lymphatic diameter. (C) Representative whole mount staining of CD31 + LYVE1‐ collecting lymphatics from young and aged mice with quantification of lymphatic diameter. (D) Confocal imaging of VE cadherin‐positive initial lymphatics and (E) RNA quantification of VE‐cadherin in hearts from young and aged mice. (F) Confocal imaging and quantification of lymphatic vessel density in the epicardium of young and aged mice. Magnified insets show LYVE1+/VEGFR‐3+ lymphatic endothelial cells (yellow upon merge). (*n* = 3 for all groups) *p < 0.05; **p < 0.01.

We have previously shown that aged C57BL/6 mice demonstrate hallmarks of heart failure with preserved ejection fraction, including adverse myocardial remodeling, impaired diastolic function, and impaired cardiac reserves (Roh et al. 2020). We found that aged 20‐month‐old C57BL/6 male mice also exhibited a significant decrease in overall LYVE‐1 staining (Figure S2) and an increase in cardiac lymphatic vessel dilation compared to young mice (Figure 3A). The epicardium of aged mice showed initial lymphatic vessel diameters more than two‐fold larger than what we observed in young mice (Figure 3B), and collecting lymphatics greater than threefold in diameter (Figure 3C). As expected, epicardial collecting lymphatic vessels were negative for LYVE‐1 but positive for Prox1 (Figure S3). Aged hearts also showed a decrease in lymphatic endothelial integrity. Vascular endothelial (VE)‐cadherin maintains endothelial cell contacts, and its loss leads to increased lymphatic permeability and leukocyte extravasation (Harris et al. 2022). Aged hearts demonstrated a marked decrease in VE‐cadherin levels in collecting lymphatics, assessed by both confocal imaging (Figure 3D) and quantitative PCR (Figure 3E). Lymphatic density in the epicardium of aged hearts was also significantly lower than in young hearts (Figure 3F).

### 3.3 Exercise Training Remodels Lymphatic Vasculature of the Aged Heart

**FIGURE 4:**
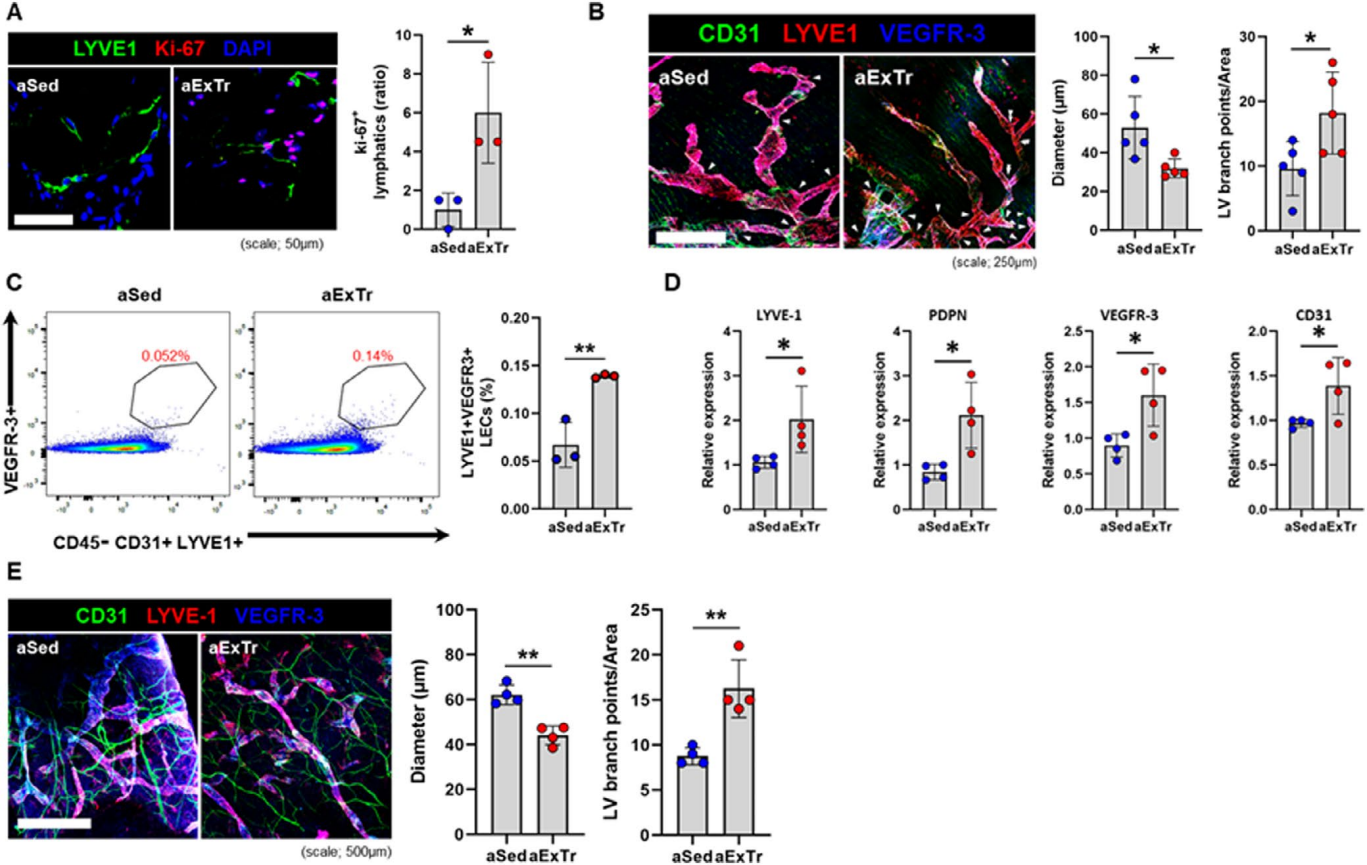
Eight weeks of exercise training enhanced lymphangiogenesis and lymphatic remodeling in the hearts of 20‐month‐old mice. (A) Confocal imaging and quantification of lymphatic vessels costained with LYVE‐1 and the proliferative marker Ki67 in the hearts of aged sedentary (aSed) control and aged exercise‐trained (aExTr) mice (*n* = 3 per group). (B) Whole mount staining for LYVE‐1+ epicardial lymphatics from aged sedentary and aged exercised mice with quantification of diameter and branching points (white arrows) (*n* = 5 per group). (C) Flow plots and quantification of lymphatic endothelial cells (CD45‐/CD31+/LYVE‐1+/VEGFR‐3+) in hearts from aged sedentary and aged exercised mice (*n* = 3 per group). (D) mRNA levels of lymphangiogenic markers in aged hearts following exercise training (*n* = 4 per group). (E) Whole mount staining of peripheral lymphatics under the ear skin in aged sedentary and aged exercised mice with quantification of diameter and branch points (*n* = 4 per group). *p < 0.05; **p < 0.01.

We have shown that 8 weeks of voluntary wheel running or forced treadmill running in older mice improve cardiac remodeling and performance (Lerchenmuller et al. 2022; Roh et al. 2020). Since the mechanisms underlying these benefits are not fully understood, we sought to determine the effects of ExTr on the adverse lymphatic remodeling seen in the aged heart. Here, we collected hearts from 20‐month‐old mice after wheel running (average daily distance per mouse = 0.97 km) to examine whether ExTr can stimulate lymphangiogenesis in the heart. Cells doubly positive for both LYVE‐1 and the proliferative marker Ki67 were found in greater numbers in exercised aged hearts than in sedentary controls, indicating that ExTr stimulated the proliferation of LECs (Figure 4A). 3D confocal images of LYVE‐1+/VEGFR‐3+ epicardial lymphatics showed that ExTr resulted in decreased diameter and increased branching (Figure 4B). Single LECs (CD45‐/LYVE‐1+/VEGFR‐3+) were isolated from whole hearts and quantified by flow cytometry. There was a significantly higher percentage of LECs in exercised hearts than in sedentary ones (Figure 4C). This was further supported by increased expression of lymphangiogenic markers LYVE‐1, PDPN, and VEGFR‐3 in mRNAs from ExTr whole hearts (Figure 4D). Not surprisingly, exercise also stimulated systemic lymphatic vasculature expansion. Peripheral lymphatics under the ear skin in aged mice following ExTr exhibited similarly decreased vessel dilation and increased branching (Figure 4E).

### 3.4 Exercise Alters the Local Microenvironment of Cardiac Lymphatics

**FIGURE 5:**
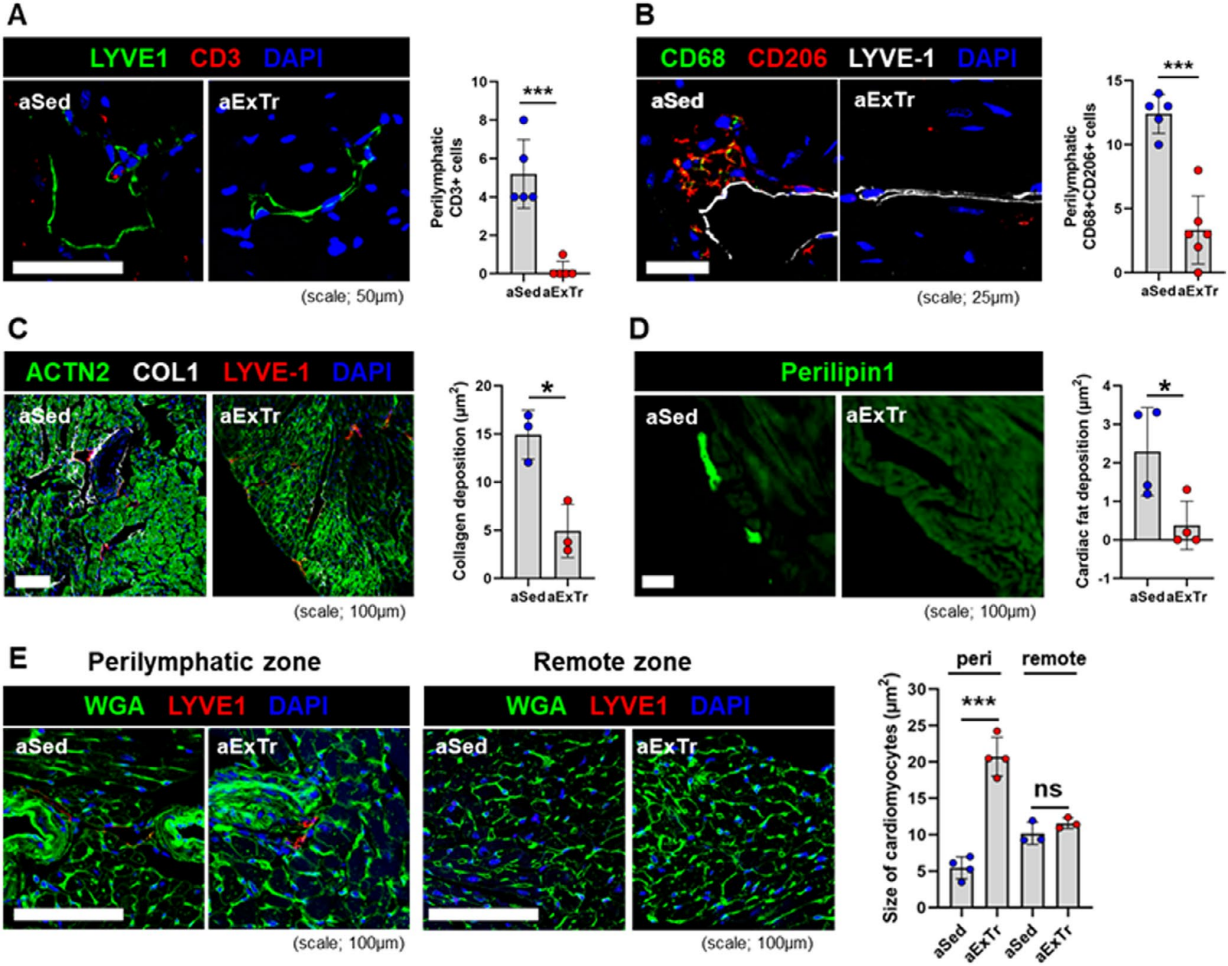
Exercise remodeled the perilymphatic microenvironment in aged hearts. (A) Confocal imaging and quantification of CD3+ T cells in peri‐lymphatic regions in the hearts of aged sedentary (aSed) and aged exercise‐trained (aExTr) mice (*n* = 5 per group). (B) Confocal imaging and quantification of CD68+/CD206+ macrophages in peri‐lymphatic regions in the hearts of aged sedentary and aged exercised mice (*n* = 5 per group). (C) Confocal imaging of collagen1, alpha‐actinin‐2, and LYVE‐1 with quantification of perilymphatic collagen (*n* = 3 per group). (D) Representative images and quantification of perilipin1+ cardiac fat in aged sedentary and aged exercised mice (*n* = 4 per group). (E) Confocal imaging of WGA‐stained cardiomyocytes and quantification of their size after exercise training in regions either adjacent to or remote from lymphatic vessels (*n* = 3–4 per group). *p < 0.05; ***p < 0.001; ns: Not significant.

Given the role of lymphatic vessels in trafficking immune cells, we next looked at different subsets of leukocytes in the aged heart under sedentary and exercised states. Wheel running resulted in dramatic decreases in CD3+ lymphocytes (Figure 5A) and CD68+/CD206+ macrophages (Figure 5B) in peri‐lymphatic regions. Interestingly, this was accompanied by exercise‐induced decreases in both fibrosis and fat deposition, as visualized by collagen‐1 (Figure 5C) and perilipin‐1 staining (Figure 5D). Finally, wheat germ agglutinin (WGA) staining of these areas adjacent to the lymphatic vessels revealed larger cardiomyocytes (Figure 5E), suggesting that improved lymphatic flow supports regional myocyte physiologic hypertrophy.

### 3.5 Exercise Improves Cardiac Lymphatic Function

**FIGURE 6:**
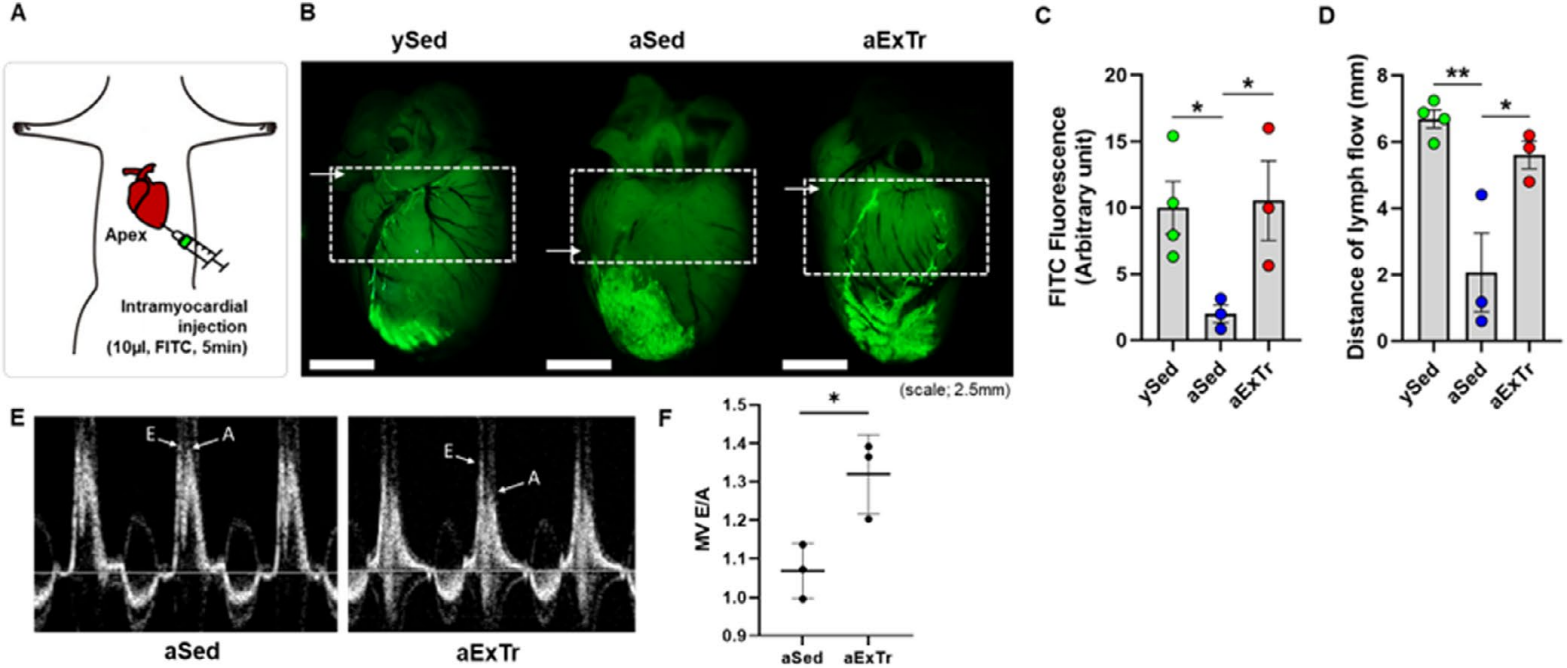
Exercise training enhanced cardiac lymphatic flow. (A) Schematic diagram of injection of fluorescent tracer (FITC‐dextran) into the apex of the beating heart. (B) Representative intravital microscopy images of epicardial lymphatic vessels perfused with FITC‐dextran in young sedentary (ySed), aged sedentary (aSed), and aged exercise‐trained (aExTr) mice. (C) Quantification of FITC fluorescence in the upper half of the heart (dotted box, panel B) and the (D) maximal distance traveled from the apex of the heart (white arrows, panel B) (*n* = 3–4 per group). (E) Representative echocardiographic waveforms showing peak velocities through the mitral valve during early (E) and late (A) diastole. (F) Mitral valve E/A ratio in aged sedentary and aged exercised mice (*n* = 3 per group). *p < 0.05; **p < 0.01.

We next used intravital cardiac lymphangiography to monitor lymphatic flow in the beating mouse heart. Aged 22‐month‐old mice underwent treadmill running for 6 weeks and were compared to their sedentary littermates. At the end of the exercise training, fluorescent tracer (FITC‐dextran) injected into the apex of beating hearts allowed visualization of epicardial lymphatics (Henri et al. 2016) (Figure 6A). Migration of the tracer was significantly impaired in aged sedentary mice versus young controls (Figure 6B). Exercise training markedly restored lymphatic flow in aged mice, as measured by both overall distribution (Figure 6C) and distance traveled (Figure 6D) by the tracer. Cardiac function was measured by echocardiography (VisualSonics, Vevo 3100) in aged‐sedentary and aged‐exercised mice prior to lymphangiography. While there were no significant differences in systolic function, aged‐exercised mice displayed transmitral flows indicative of improved diastolic function (Figure 6E,F).

## 4 Discussion

Several reports have suggested that cardiac lymphatic growth and remodeling could be a novel therapeutic target to improve cardiac function in different injury and disease states (Aspelund et al. 2016; Cui 2010; Dashkevich et al. 2016; Henri et al. 2016; Ishikawa et al. 2007; Kholova et al. 2011; Klotz et al. 2015). For example, cardiac lymphatics help orchestrate immune cell trafficking around the site of injury after myocardial infarction (MI) (Nahrendorf et al. 2007), and cardiac lymphangiogenesis activated by AAV‐guided expression of VEGF‐C helps resolve peri‐infarct inflammation (Houssari et al. 2020). Importantly, VEGF‐C treatment leads to less wall thinning caused by MI and to significant improvement in cardiac function (Klotz et al. 2015). Indeed, the LEC‐secreted protein reelin (RELN) has been shown to direct cardiac proliferation and survival (Liu et al. 2020).

Aging is a significant risk factor for peripheral lymphatic impairment, where decreased glycocalyx and intercellular junctions, increased permeability, and reduced lymph transport and pathogen clearance are observed. Aging‐associated lymphatic dysfunction has been described in multiple tissues such as the thoracic duct and skin (Akl et al. 2011; Gasheva et al. 2007; Ma et al. 2017; Rustenhoven et al. 2023; Yoon et al. 2024). In this study, we have now characterized cardiac lymphatic vasculature during aging and discovered similar defects as those found in other organs.

Exercise in young mice increases the expression of the LEC markers VEGFR‐3 and Prox1 in the heart, and a higher density of LYVE‐1 and podoplanin double‐positive lymphatic vessels (Bei et al. 2022). Vascular endothelial growth factor (VEGF)‐C and ‐D are principal drivers of lymphangiogenesis via the receptor VEGFR‐3 and are elevated in the heart after exercise (Bei et al. 2022). Interestingly, lymphangiogenesis seems to promote and be required for physiological hypertrophy. Conditioned media from LECs stimulate cardiomyocyte hypertrophy and proliferation (Brutsaert 2003; Hsieh et al. 2006). Treatment of mice with SAR131675, a VEGFR‐3 inhibitor, attenuates not only the exercise‐induced increase in lymphatic vessel density but cardiac growth as well (Bei et al. 2022).

Here, we show that cardiac lymphatic vasculature undergoes a process of adverse remodeling with aging, characterized by a decrease in lymphatic vessel density, compensatory dilatation, and increased permeability. Furthermore, impaired lymphatic function can lead to persistent inflammation and fibrosis. Indeed, aged hearts showed greater infiltration of immune cells, including lymphocytes and macrophages, into the myocardium, as well as increased accumulation of collagen and reduced size of cardiomyocytes in peri‐lymphatic regions. Importantly, we found that 8 weeks of voluntary wheel running promoted cardiac lymphangiogenesis, demonstrated by elevated levels of VEGFR‐3, lymphatic sprouting, and markers of proliferation. Exercise decreased lymphatic dilation and permeability and restored lymphatic flow as measured by intravital lymphangiography. Exercise training inhibited peri‐lymphatic inflammation, lipid deposition, and fibrosis and promoted physiological cardiomyocyte hypertrophy. It is tempting to speculate that exercise would exert beneficial effects in other conditions of chronic peri‐lymphatic inflammation or fibroproliferation. For example, excessive lipid and cholesterol absorption in mouse models of obesity leads to reduced peripheral lymphatic pumping and smaller lymph nodes (Angeli et al. 2004; Blum et al. 2014; Hespe et al. 2016; Lim et al. 2009; Savetsky et al. 2015; Weitman et al. 2013; Zawieja et al. 2012). Exercise may very well protect against dysregulated lymphatic function and inflammation in the heart in the context of such metabolic disorders. Exercise remains the most effective lifestyle intervention in older adults with heart failure (Kitzman et al. 2021; Roh et al. 2016). While the mechanisms underlying its benefits are not entirely clear, it is certainly plausible that they could be mediated, at least in part, through improving cardiac lymphatic function.

Limitations of our study include using only male mice thus far, as biological sex may influence cardiac lymphatics. Our studies, while detailing the molecular, histological, architectural, and functional changes of cardiac lymphatics during aging, have not fully explored the molecular mechanisms of exercise‐induced remodeling. Mechanistic studies to characterize the interaction between aging and lymphatic changes, with a special focus on lymphangiogenic (e.g., VEGF‐C) or lymphangiocrine factors, are of great interest in ongoing studies. Furthermore, single‐cell gene expression patterns of aged LECs in the heart before and after exercise training may reveal novel mediators of lymphatic remodeling. Emerging data reveal heterogeneity between LECs in different organs that allows tissue‐specific molecular adaptations to the local microenvironment (Alitalo 2011; Petrova and Koh 2018; Tammela and Alitalo 2010). For example, meningeal lymphatics are essential in maintaining brain homeostasis by draining both interstitial fluid and cerebrospinal fluid, with these and glial‐derived “glymphatics” serving as a major route for macromolecule uptake and waste clearance (Ahn et al. 2019; Da Mesquita et al. 2018; Iliff et al. 2012). Future efforts will focus on the interplay between cardiac LECs, lymphatic muscle cells, and the surrounding myocardium in both male and female mice. This will provide a deeper understanding of the cardiac‐specific roles of lymphatics in maintaining metabolic, fluid, and immune homeostasis in the heart.

## Author Contributions

K.R. and J.R. designed the study. K.R., H.L., R.N.F., L.Z., C.Z., S.S., P.X., C.S., and Y.Z. conducted the experiments. K.R., H.L., J.R.B.G., A.L., S.K., T.P.P., A.A., N.H., J.D.R., F.I., R.M., and A.R., performed data analysis and interpretation. K.R. and J.R. wrote the manuscript with contributions from all authors.

## Conflicts of Interest

J.D.R. receives support from Amgen, Keros, and Genentech, along with the following patents (11,834,508, WO2018175460A1, US20180193529A1). J.R. consults for Takeda Neurosciences. All research support, patents, and consultancy are unrelated to this work.

## Supporting information


**Figure S1.** Representative intravital microscopy videos of PLVs perfused with FITC‐dextran from sedentary (Sed) and exercise‐trained (ExTr) 2‐month‐old mice.

**Figure S2.** LYVE‐1 staining of cross sections of young and aged hearts.

**Figure S3.** Epicardial staining of Prox1‐eGFP mouse heart.

**Table S1.** Primer sequences used for quantitative PCR.

## Data Availability

Data sharing not applicable to this article as no datasets were generated or analysed during the current study.
