# Supplementary figures and images for: Exercise‐Induced Cardiac Lymphatic Remodeling Mitigates Inflammation in the Aging Heart
Source: Aging Cell. 2025 Mar 13;24(6):e70043. doi: 10.1111/acel.70043 (PMC12151892; doi:10.1111/acel.70043)

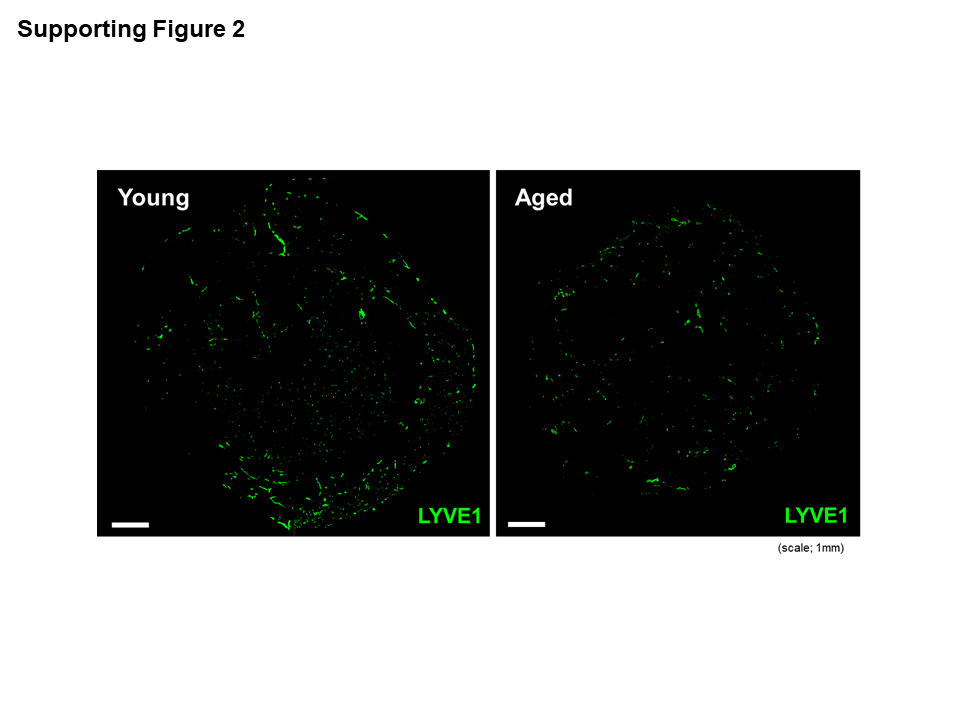

Supplement: Supplementary file 2 — Figure S2. LYVE‐1 staining of cross sections of young and aged hearts. [file ACEL-24-e70043-s004.tif]

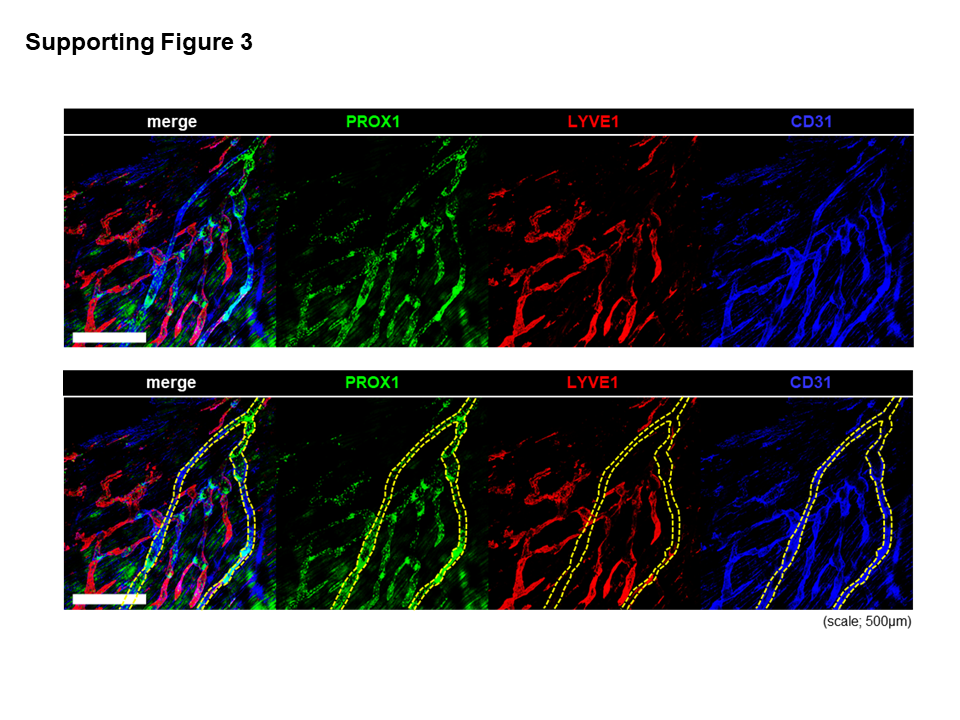

Supplement: Supplementary file 3 — Figure S3. Epicardial staining of Prox1‐eGFP mouse heart. [file ACEL-24-e70043-s002.tif]

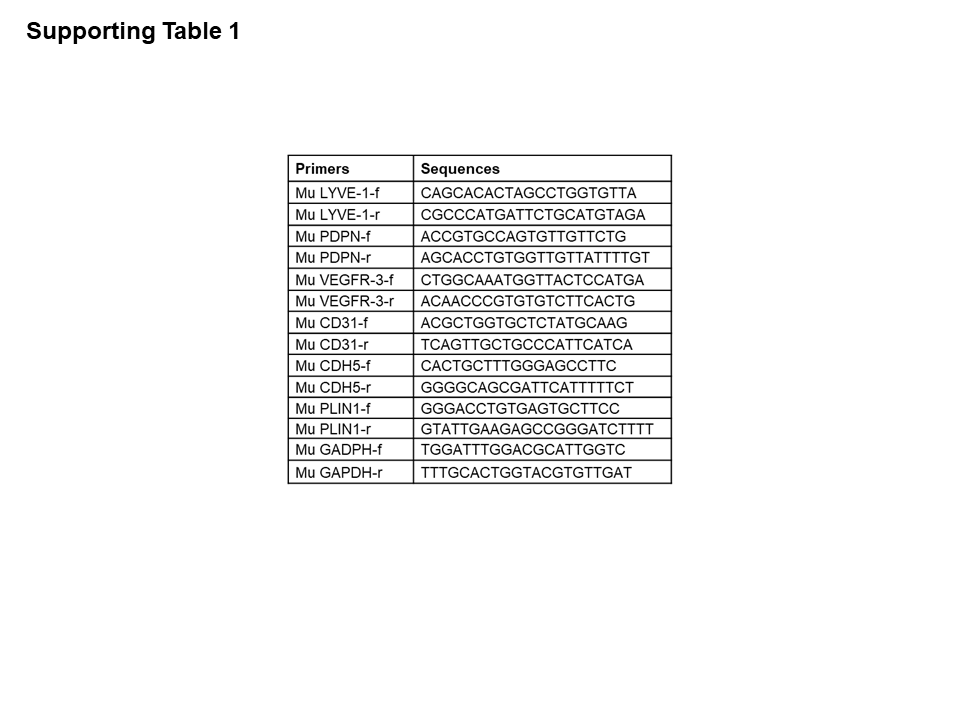

Supplement: Supplementary file 4 — Table S1. Primer sequences used for quantitative PCR. [file ACEL-24-e70043-s003.tif]
